# Mineral analysis reveals extreme manganese concentrations in wild harvested and commercially available edible termites

**DOI:** 10.1038/s41598-020-63157-7

**Published:** 2020-04-09

**Authors:** Rudi L. Verspoor, Murielle Soglo, Razack Adeoti, Rousseau Djouaka, Sam Edwards, Rikard Fristedt, Maud Langton, Rosana Moriana, Matthew Osborne, Catherine L. Parr, Kathryn Powell, Gregory D. D. Hurst, Rikard Landberg

**Affiliations:** 1 Institute of Integrative Biology, University of Liverpool, Liverpool, L69 7ZB United Kingdom; 2 IITA Research Station, Godomey, Benin; 3 Chalmers University of Technology, Department of Biology and Biological Engineering, Division of Food and Nutrition Science, SE-412 96 Göteborg, Sweden; 4 Swedish University of Agricultural Sciences, Department of Molecular Sciences, Box 7015, 750 07 Uppsala, Sweden; 5 Stockholm Environment Institute, Stockholm, Sweden; 6 School of Environmental Science, University of Liverpool, Liverpool, L69 3GP United Kingdom; 7 Department of Zoology & Entomology, University of Pretoria, Pretoria, South Africa; 8 School of Animal, Plant and Environmental Sciences, University of Witwatersrand, Wits, South Africa

**Keywords:** Entomology, Environmental chemistry

## Abstract

Termites are widely used as a food resource, particularly in Africa and Asia. Markets for insects as food are also expanding worldwide. To inform the development of insect-based foods, we analysed selected minerals (Fe-Mn-Zn-Cu-Mg) in wild-harvested and commercially available termites. Mineral values were compared to selected commercially available insects. Alate termites, of the genera *Macrotermes* and *Odontotermes*, showed remarkably high manganese (Mn) content (292–515 mg/100 gdw), roughly 50–100 times the concentrations detected in other insects. Other mineral elements occur at moderate concentrations in all insects examined. On further examination, the Mn is located primarily in the abdomens of the *Macrotermes subhyalinus*; with scanning electron microscopy revealing small spherical structures highly enriched for Mn. We identify the fungus comb, of *Macrotermes subhyanus*, as a potential biological source of the high Mn concentrations. Consuming even small quantities of termite alates could exceed current upper recommended intakes for Mn in both adults and children. Given the widespread use of termites as food, a better understanding the sources, distribution and bio-availability of these high Mn concentrations in termite alates is needed.

## Introduction

Insects are consumed as food in many countries around the world. Much of this consumption derives from cultural traditions of entomophagy, particularly in Africa, south-east Asia, and central America^1,2^. Marketing and export of edible insects can also provide an important source of revenue (e.g.^3^) and the use of insects as food is expanding into countries beyond those where use is traditional. As a result, edible insects now attract global attention in research, media and commercial sectors; particularly with respect to their contribution to food security and sustainability^4,5^.

The expanding market of edible insects creates challenges in terms of regulation and quality control^6^. Novel edible insects are reaching new markets, bringing unique obstacles for value chain regulation; for example accurate identification of species. Specific factors, such as wild harvesting and rural processing bring additional difficulties when trying to establish and maintain the quality of insect foods^6,7^; for example, the accumulation of heavy metals^8,9^. However, the extent and source of variations in mineral content within and between species remains largely unknown. This knowledge gap is important, as nutritional information underpins food safety standards and is vital for decision making when novel foods are entering markets, for example in the European Union^10^.

Termites, in particular winged termites (henceforth referred to as ‘alates’), are widely consumed in quantity as food across Africa, America and Asia^1,11,12^ when they emerge *en masse* during the rainy season. Worldwide it is reported that 43 species are used as either human or livestock feed, with some species, particularly those from the genus *Macrotermes*, most commonly used as human food^12^. A number of studies on alates as food have highlighted their nutritional value and potential contribution to food security, both in raw and processed form^13–15^,due to the high protein and fat content of these insects^16,17^. Alates are also available in local and international markets, which provides local income and contributes to economic development^16–18^. In contrast to many farmed insects, alates are wild harvested, which could result in greater differences between collections due to variation in diets, the species collected, and the local conditions. In some insects, including edible species, accumulation of minerals to toxic levels has been associated with environmental contamination^8,9,19^. In addition, there is a startling variation in trace minerals concentrations reported in studies examining alates (Table 1). Establishing consistent estimates of mineral concentrations in alates is critical when assessing their potential benefit and informing their potential marketability^20,21^.

**Table 1:** Example of highest and lowest reports of mineral contents for termites of the genus *Macrotermes*.

Level reported	Fe	Zn	Cu	Mn	Mg
Lowest	0.14 (26)	0.21 (26)	0.03 (26)	0.08 (13)	0.15 (14)
Highest	116 (14)	15 (18)	5 (18)	714 (18)	81 (18)
RDA (Child 4–8)	10	5	0.44 *	1.5 *	130
RDA (Adult female)	18	8	0.9 *	1.8 *	320
RUL (Child 4–8)	40	12	3	3	110**
RUL (Adult female)	45	40	10	11	350**

Values are reported as mg/100g fresh weight. Numbers in parentheses indicate the studies referenced. Dietary advice values are presented as recommended daily allowance (RDA), and the recommended Upper Limit (RUL) in mg/day^20,21^. *values represent adequate intake (AI). **refers to magnesium in supplement form.

We examine the content of five trace minerals (Fe-Mn-Zn-Cu-Mg) in a selection of alates from Benin and South Africa, where termites are commonly consumed as human food. We compare these concentrations of trace minerals to commercially available insects, including alate termites.

## Methods

### Insect material used for analysis

#### Field collection of edible termites

**Figure 1:**
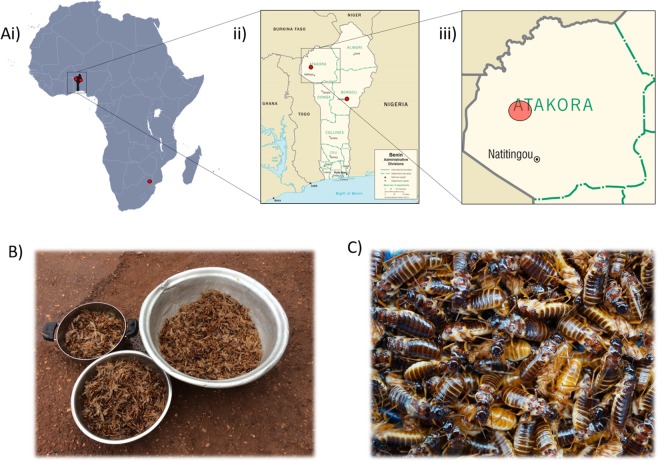
(**A**) (i) The locations of broader geographic sampling (ii) The sampling sites of Macrotermes spp. Alates within Benin and (iii) The zone in which more intensive sampling of Macrotermes spp. alates was carried out. (**B**) An image of alate termites just after collection prior to de-winging. (**C**) An image of a de-winged Macrotermes subhyalinus alates.

Alates of *Macrotermes subhyalinus* were collected from north-west Benin, where Macrotermes termites are consumed as food^22^. Termites were identified to species using classical taxonomy of termite soldiers collected from mounds in the area. Fungus comb, mound soil, and termite soldiers were also collected using a hoe from a subset of the mounds visited in the area around Tanguieta. The samples were collected by hand around lights at dusk into a basin of clean water, between May and August 2017 and 2018. The locations were within a 10 km radius of Tanguieta, Atakora department, Benin (Fig. 1Ai; Table S1). Samples therefore constitute a combination of alate termites from multiple mounds in a given area. We also collected samples of the large tobacco cricket (*Brachytrupes membranaceus*) from the same region in north-west Benin, by digging them out from their burrows using a hoe, to provide a second local edible insect species. Two further samples of alates were also collected from additional sites at Parakou (Borgou department, Benin), and from near Acornhoek (Mpumalanga, South Africa; Fig. 1A). In both these sampling locations termites are by communities. All samples were rinsed with clean bottled water after collection to remove any mud or dust. Termites were then briefly sun-dried to remove external moisture and to assist with wing removal, a treatment that is traditional in Benin. Following wing removal then stored in food-grade storage containers at −20 °C or below until mineral analysis. For samples collected from South Africa, de-winged termites were oven dried at 60° overnight to allow for further transport at room temperature.

#### Commercial insect types used

A selection of dried, processed edible insects were purchased from a supplier in the UK to provide a product comparison for the wild harvested termites (Table S1). The commercial insects included both farmed insects and wild-harvested insects (including one sample of alate termites), multiple insect orders, and many widely consumed insect species. In particular, analysing commercially available alate termite and leaf-cutter ant queens provided a suitable reference comparison for our termites, and more widely to insects that use fungus farming for food. Single packets of each insect type were purchased and tested. To confirm the identity of the species being examined insects were barcoded^23^.

### Extraction and measurement of trace minerals

#### Sample transport and analysis

All mineral analysis was conducted at Chalmers University of Technology, Gothenburg unless stated otherwise. All termite samples from Benin were stored and transported fresh at −20 °C. Samples collected from Acornhuek in South Africa were dried and transported at room temperature. All commercial insects are delivered pre-dried and were then frozen and transported at −80 °C to Sweden from the UK. Upon arrival in Sweden all samples were stored at −80 °C.

#### Moisture and total ash content for termite samples

Four replicates of fresh termites from north-west Benin were measured for total moisture and total ash content. To this end, 50 g of termites (de-winged) were freeze dried for a period of 72 hours. This was not possible to do with the South Africa termite samples or the commercial insect samples as they arrived pre-dried. Total ash content was then determined according to AOAC Official Method 942.05.

#### Extraction of minerals from insect material

The fresh termite samples collected from Benin were freeze-dried before mineral extraction. After drying, for all insects between 25 and 100 individuals were homogenised in a food grinder in order to obtain 2 g of ground insect material. In total, 150–300 mg of sample was then used for mineral extraction. Microwave assisted acid digestion was performed in reinforced Teflon tubes using 3 ml milliQ H_2_0, 750 µl nitric acid and 150 µl HCl (Fisher Chemical, Sweden). Samples were ramped to 180 °C, held at 180 °C for 30 minutes, and left to cool to room temperature. Following extraction all mineral samples were made up to 10 ml with milliQ water and stored for further dilution and quantification.

#### Quantification of trace minerals

Minerals (Fe-Mn-Zn-Cu-Mg) were quantified using atomic absorption spectroscopy on an Agilent Technologies 200 Series AA 240FS AA with an UltrAA Boosted Lamp Supply with Agilent hollow cathode lamps. An average was calculated from triplicate repeat measures of absorption area (2.5 second time period, 6 second pre-read delay). Lamp position was optimised manually prior to each run. Dilutions of standards were prepared and used to calibrate biological samples, as advised for the Agilent AAS 200 series per operating protocol.

The discovery of unexpectedly high manganese (Mn) content in termites led to two further analyses of Mn concentrations in alates from north-west Benin. Mn was examined using two additional independent methods. Mn was examined using ion chromatography^24^ and also independently validated by the National Food Agency in Sweden using ICP-MS, who additionally assayed lead, aluminium, molybdenum and cadmium concentrations. Six termite samples were analyzed for Mn along with Al, Fe, Cu, Zn, Mo, Cd and Pb by an accredited ICP-MS method at the National Food Agency in Uppsala Sweden. Samples were microwave extracted using nitric acid and hydrochloric acid at 200 °C. The method used was based on NMKL method nr 186 and EN 15763:2009. Due to high concentrations of Mn in the samples, dilutions were necessary and therefore several metals were not quantified by accreditation.

To test for potential sources of the high Mn concentrations in alates, Mn content in soil samples from *Macrotermes* mounds and fungus comb samples from within mounds were also examined using the atomic absorption spectroscopy (three biological replicates respectively).

#### Distribution of trace minerals in termites

Termite samples were mounted on carbon stubs for examination in a HITACHI TM-1000 Scanning Electron Microscopy with an energy-dispersive X-ray spectroscopy (EDX) detector (Oxford Instruments). EDX detector specifically detect major elements (above the atomic number of Na) placed in a specific area of the SEM picture. The distribution of the Mn in the integument was assessed by examination of the whole termite. The termites were further dissected to evaluate the distribution of the minerals in the interior of the thorax and abdomen.

#### Statistics

The concentrations of Mn were compared: alates against soldiers, abdomens vs cephalothoraxes, and mound soil versus fungus comb using t-tests after log transformation of data. Calculations were performed in R^25^. All values for commercial insects are reported in mg/100gDM (dry matter) as commercial insects were delivered pre-dried.. We also report our values for alates from north-west Benin in mg/100gfw (fresh weight).

## Results

### Mineral contents found in wild-harvested and commercial insects

Alates collected from Benin contained 52.5% ± 1.2_SE_ water of which 3.65% ± 0.27_SE_ was ash. There was considerable variation between different insects for all minerals examined (Table 2). The most striking result was the high Mn content found in alates. Alates from Benin, South Africa, and commercial alates purchased online all had extremely high concentrations of Mn (271–515 mg Mn/100gdw). These concentrations are around 100 fold more than we found in other commercial insects, (range: 0.5–3.9 mg Mn/100gdw). Both the secondary ion-chromatography testing for Mn concentrations (388 mg Mn/100gdw) and the independent ICP-MS evaluation (489 mg Mn/100gdw) confirmed the high Mn content in north-west Benin alates (Table S2). Other heavy metal values (Mb-Cd-Pb) were either at very low levels or below the detectable range (Table S2).

**Table 2:** The quantities of five minerals found in different insect species.

Insect type	Location	Fe	Zn	Cu	Mn	Mg
Alate termites (*Macrotermes subhyalinus*)	Tanguieta, Benin	13.4 ± 0.4	10.3 ± 0.4	8.5 ± 0.5	422 ± 27	104.8 ± 6.7
		***6.2***^**ɵ**^	***4.9***^**ɵ**^	***4.0***^**ɵ**^	***200.5***^***ɵ***^	***39.8***^***ɵ***^
Alate termites (*Macrotermes* spp.)	Parakou Benin	10.3 ± 0.3^**ʈ**^	13.8 ± 0.3^**ʈ**^	8.2 ± 0.3^**ʈ**^	292.7 ± 21.4^**ʈ**^	Not measured
Alate Termites (*Odontotermes* spp.)	South Africa	8.8 ± 0.2^**ʈ**^	9.2 ± 0.4^**ʈ**^	6.6 ± 0.4^**ʈ**^	515 ± 74^**ʈ**^	Not measured
Alate Termites (*Macrotermes* spp.)	South Africa	9.8 ± 0.5^**ʈ**^	12.0 ± 0.4^**ʈ**^	5.1 ± 0.6^**ʈ**^	481 ± 112^**ʈ**^	Not measured
Alate termite (*Odontotermes spp*.)^*^	South-East Asia	13.9 ± 0.5^**ʈ**^	12.9 ± 0.3^**ʈ**^	7.6 ± 0.3^**ʈ**^	271.4 ± 29.8^**ʈ**^	95.0 ± 1.3^**ʈ**^
Tobacco Cricket (*Brachytrupes membranaceus*)	Tanguieta, Benin	65.7 ± 3.1^**ʈ**^	16.6 ± 0.6^**ʈ**^	1.0 ± 0.1^**ʈ**^	2.8 ± 0.2^**ʈ**^	Not measured
Locust (*Locusta migratoria*)^a^	UK bought pre-dried	9.2 ± 0.5^**ʈ**^	25.0 ± 0.2^**ʈ**^	6.0 ± 0.2^**ʈ**^	1.0 ± 0.1^**ʈ**^	85.0 ± 1.2^**ʈ**^
House cricket (*Acheta domesticus*)^a^	UK bought pre-dried	9.2 ± 0.6^**ʈ**^	26.6 ± 0.8^**ʈ**^	5.3 ± 0.1^**ʈ**^	3.8 ± 0.3^**ʈ**^	68.1 ± 0.4^**ʈ**^
Water scorpion (*Lethoserus indicus*)^a^	UK bought pre-dried	33.4 ± 0.1^**ʈ**^	11.5 ± 0.1^**ʈ**^	2.3 ± 0.1^**ʈ**^	1.2 ± 0.1^**ʈ**^	111.3 ± 2.8^**ʈ**^
Queen leafcutter Ant (*Atta* spp.)^a^	UK bought pre-dried	11.0 ± 0.3^**ʈ**^	19.0 ± 0.8^**ʈ**^	2.8 ± 0.1^**ʈ**^	2.1 ± 0.5^**ʈ**^	64.6 ± 2.6^**ʈ**^
Mopane worm *(Gonimbrasi belina)*	UK bought pre-dried	54.5 ± 3.6^**ʈ**^	16.6 ± 0.6^**ʈ**^	6.4 ± 0.2^**ʈ**^	3.9 ± 0.2^**ʈ**^	Not measured
Silkworm pupae (*Bombyx mori*)^a^	UK bought pre-dried	3.8 ± 0.1^**ʈ**^	17.7 ± 0.2^**ʈ**^	2.2 ± 0.1^**ʈ**^	1.9 ± 0.1^**ʈ**^	305.5 ± 3.1^**ʈ**^
Mealworm (*Tenebrio molitor*)^a^	UK bought pre-dried	6.0 ± 0.2^**ʈ**^	14.4 ± 0.2^**ʈ**^	2.5 ± 0.1^**ʈ**^	0.5 ± 0.1^**ʈ**^	244.6 ± 2.9^**ʈ**^

All values are expressed as mg/100gdw material and the variation is the SEM. ^**ʈ**^ means are calculated from triplicates of a single quantity of termites purchased or collected from supplier. Values in bold denoted by ^ɵ^ mg/100gfw for termite alates collected from north Benin. ^a^ Indicates as identified by barcoding of commercially supplied insects ±SE.

Particularly high concentrations of magnesium were found in two commercial insects, mealworms and silkworm pupae (244.6 and 305.5 mg Mg/100gdw respectively), which is approximately 3–5 times the concentrations measured in the other insects. Also of note were the relatively high concentrations of iron found in the water scorpions, the large tobacco cricket, and mopane worms (33.4, 65.7 and 54.5 mg Fe/100gdw respectively), approximately 2–3 times the concentrations measured in the other insects. The locust, house cricket and large tobacco cricket showed the highest quantities of zinc, and termites and water scorpions the lowest (Table 2).

### Potential sources of manganese levels in termites

To explore the origin of the high Mn in termites, we first compared soldier and alate castes of *M. subhylanus* from north-west Benin. Termite soldiers contained significantly lower Mn concentrations (14.6 mg Mn/100gdw) than were detected in the alates (422.2 mg Mn/100gdw) (t = 30.3 df = 4.4 P < 0.01; Table 3). Soldier termites did however, still show slightly higher Mn concentrations than other commercial insects. To establish if the Mn was anatomically localised and could be reduced by processing (for example beheading of alates prior to consumption) we examined whether heads and bodies of *M. subhylanus* differed in Mn content. Termite heads had significantly lower Mn concentrations than the abdomen material (t = 27.2 df = 3.3 p < 0.01; Table 3), with about 200 times the concentration of Mn found in the abdomen compared to the head.

**Table 3:** The quantities of manganese found in different termite castes (soldiers and alates), different parts of alate anatomy (cephalothorax or abdomens only), and two components of the termite mound (external soil and comb from the fungus gallery).

Factor compared	Description	Manganese (mg/100gdw)
Termite caste	Alates (n = 9)	423 ± 27
	Soldiers (n = 3)	15 ± 1
Alate body part	Heads only (n = 3)	3 ± 1
	Abdomens only (n = 3)	649 ± 63
Mound component	External soil (n = 3)	0.04 ± 0.01
	Comb from fungus gallery (n = 3)	342 ± 42

Values are expressed as mg Mn/100gdw ± SEM. *Macrotermes subhyalinus* specimens from Benin.

Further comparison of *Macrotermes* mound soil samples with fungus comb samples also showed large differences in levels of Mn. Fungus combs were found to have significantly higher Mn concentrations than mound soil (t = 26.9, df = 2.5, P < 0.01; Table 3), with 342 mg Mn/100gdw and 0.04 mg Mn/100gdw respectively.

**Figure 2:**
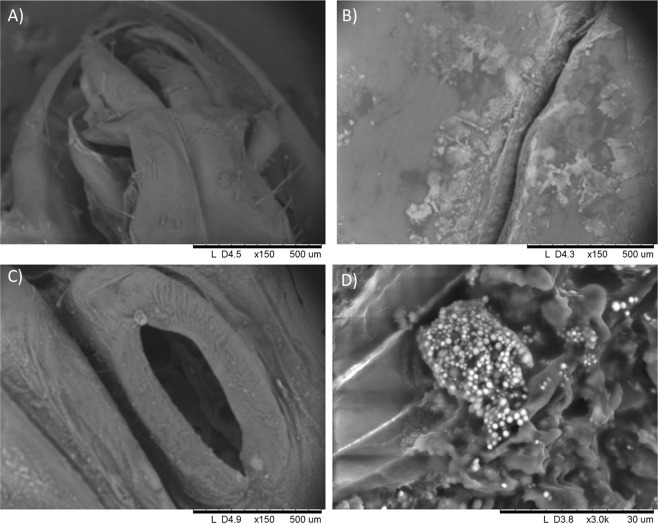
Scanning Electronic Microscopy images used to evaluate the distribution of Manganese in different parts of termite alates: (**A**) mandibles, (**B**) cuticle, (**C**) spiracle and (**D**) interior of the abdomen.

To follow up to the detection of high Mn found localised to the abdomens of *M. subhylinus* we performed scanning electron microscopy (SEM) imaging. The SEM-EDX area analysis on the external cuticle of an alate shows moderate Mn in the mandibles (Fig. 2A; Table 4) and abdominal cuticle (Fig. 2B; Table 4), with more enrichment of Mn in the spiracle (Fig. 2C; Table 4; Table S3). Hotspots of Mn within the termite abdomen were observed (Fig. 2D; Table 4). Spherical structures with heterogeneous diameters (ranging from 0.5 to 2 µm) are placed together in a specific area of around 15 × 18 µm in the abdomen.

**Table 4:** Composition in wt% of the total elemental content for five mineral (Mg, Mn, Fe, Zn and Cu) for mandibles, abdominal cuticle, spiracle and interior abdomen of *Macrotermes subhyalinus* specimen from Benin.

	mandibles	abdominal cuticle	spiracle	interior abdomen
**Mg** (%)	n.d	0.7	1	0.7
**Mn** (%)	1.4	4.3	12	9.2
**Fe** (%)	6.5	n.d	1.6	2.8
**Zn** (%)	2.5	2.5	n.d	n.d
**Cu** (%)	n.d	n.d	n.d	n.d

## Discussion

Wild foraged termites, which represent a commonly used food resource in sub-Saharan Africa, were analysed for mineral content. These data were compared to other commercially bought insect material sold for human consumption. Our results highlight the need for systematic nutrient analysis of insects aimed for human consumption.

### High manganese specific to termites

Our most striking result is the unexpectedly high Mn content we found in all alate termites from all locations sampled, whether field collected or commercially-bought in the UK. Quantities of Mn were more than 100-fold greater than in other commercial insects. These high concentrations of Mn are in contrast to a number of studies examining other *Macroterme*s termites^14,26^, but are consistent with two previous studies from Zimbabwe and Zambia^18,27^. Such unexpected and large variation in Mn between studies could be either methodological or biological and should be the focus of a concerted review of Mn across termite species. Nonetheless, repeated findings of high concentrations of Mn suggest that certain alate termites could have a general propensity to contain high Mn. Considering alates are widely consumed^11^, and sometimes in large quantities, these data could have important implications for food safety and nutrition. We are confident the high Mn detected in these species is biologically derived and not contamination from sampling methods or materials used. First, water used during collection was bottled drinking water and so very unlikely to be a contaminant, and second, the results from the electron microscopy imaging and the specificity of the Mn to *M. subhyalinus* abdomens are both consistent with the Mn being stored within biological tissue.

Mn is a required mineral element in small quantities, but can be toxic at high levels causing the neurological condition known as manganism^28^. Animal studies indicate high levels may also be teratogenic^29^. The levels of Mn we report would mean that 100 g of dry termites could provide ~40 times the recommended upper limit (RUL) for adults (18; Table 1). The safe limit for young children (24; Table 1), who commonly consume termites, is much lower: for a 5–13 year old more than 2–5gfw termites per day (15–43 termites) will exceed the RUL^21^. A further consideration relates to the potential for competition between iron and Mn for absorption^30^. This may compound problems associated with high Mn concentrations, in particular for women and children in lower income areas where anaemia is a problem and iron deficiency is widespread. The boom-bust pattern of consumption that occurs when alates emerge *en masse* could also enhance potential effects; with large emergences being consumed immediately, thus creating acute exposures.

While the majority of reports of Mn toxicity are of people working in smelters exposed to large amounts of Mn, environmental sources such as contaminated water are also implicated^31,32^. Nonetheless, Mn poisoning has never been reported from normal dietary intake. However, we know of no study examining alates as food that looks at any effects of high Mn. In light of the concentrations of Mn that our study and other reports^18,27^, more focussed research is critical to understand why and how such high Mn concentrations are reached in alates and why different studies have reported such variable results. It also remains unclear to what extent this Mn is bioavailable when consumed by humans and in what quantity and form it is stored within the termites. Cell culture absorption assays or animal absorption assays for trace metals provide a potential route to explore this^33^. Such methods have been used to examine the bioavailability of dietary minerals in other insects and found high bioavailability when compared with other animal sources^34^. A further point of interest would be to investigate if different trace minerals from insects compete with each other during absorption, for example does the presence of high Mn impede the absorption of dietary iron.

These results may also have implications beyond just the human food chain. A recent review of Mn in insects found that high levels can have negative effects in bees and flies^35^. Our results are consistent with alate termites accumulating and storing manganese in their abdomens. However, any suggestions with respect to the underlying biology of this accumulation and if it has benefits or costs to alate termites is speculative. Alate termites also form a seasonal part of the diet for a range of animals; including insects, reptiles, amphibians, birds, and mammals. Some of these groups, for example mammals, are known to be sensitive to Mn accumulation^35^. It follows that the consumption and absorption of termites that are very rich in Mn could have downstream implications for community food webs and is worth further investigation.

We found high Mn levels in two termite genera that are regularly consumed (*Macrotermes* and *Odontotermes*). It is likely not a coincidence that both of these genera are fungus-growers and Mn concentrations were very high in the fungus comb. If fungus growing is found to be the single determinant of Mn concentrations, it remains possible that levels are lower in non-fungus growing species. A concerted effort to examine a taxonomically diverse range of edible termites (of various castes), with consistent and repeatable results, could provide a robust estimate of mineral concentrations across termites. This scale of evaluation has been repeatedly called for in reviews of insects as food, but has yet to be realised^7,18,36^.

### Sources of manganese and potential roles in alates

To further understand the high Mn concentrations in alates, we compared alates to soldiers (distinct termite castes). Soldiers and alates can both be used as food, although alates are available in larger abundances during their emergences. Previously reported enrichment of manganese in the mandibles of soldiers^37^ is one explanation for high manganese in these species. However, we found no support for the mandible-specific enrichment hypothesis: the comparison between soldiers and alates showed manganese concentrations are higher in alates and comparison between the termite cephalothorax and abdomen showed Mn enrichment was specific to the abdomen. The finding of specific Mn enriched structures within the abdomen (Fig. 2D) point instead to a biological role of manganese rich tissue. As the alate abdomen constitutes a large proportion of the total insect, removal of the abdomen would result in the majority of beneficial nutrients being lost. Thus, simple processing (e.g head removal) that is carried out in many insects^38^ is also not appropriate for reducing manganese concentrations in these alates.

Macrotermitinae termites, which include *Macrotermes*, farm fungus to breakdown plant material into digestible food. A comparison of the fungus comb and mound soil revealed remarkably high Mn concentrations in the fungus comb but not the soil. This result is consistent with an already established biological role for Mn in lignin digestion; Manganese peroxidase is one enzyme produced by basidiomycetes fungi to break down lignin^39^. In addition, Alates from some *Macrotermes* species transport fungus when founding new colonies^40,41^. Further examination of the composition of the Mn rich nodules in the alates abdomens will help explore this hypothesis. Alternately, alates may have high concentrations on Mn if it is necessary for the production of worker and soldier castes during colony formation. This may explain the high concentrations found in the commercially available alate termites labelled as *Nasutitermes*, which do not farm fungus.

### Consumption and marketing of termites

Insects can have a high economic value and could provide a means of mitigating against food insecurity^42^. However, the observed high levels of Mn could present a significant challenge to future development of termite alates if this phenomenon is widespread, particularly as a commercial product (for example 16).

From a European perspective, the approval for insects as food should be straightforward given that legislation stipulates that toxicological levels of food are expressed in terms of an absolute and binary Acceptable Daily Intake (ADI) level: if this is exceeded, then the food is not considered fit for human consumption^43^. Given this framing, currently we suggest there is little potential for the development of an export market for termite alates in an unprocessed form.

From the perspective of local consumers in Benin, the ethical calculation is more complex^44^. Many consumers in Benin live in higher risk environments where simplistic choices about what is a ‘good’ or ‘bad’ food is less realistic^45,46^. Whether to exploit an available food source for immediate nourishment or not if it may increase the risk of future illness is therefore not a simple choice. Nonetheless, we urge that the findings of this study should be brought to the attention of current consumers of termite alates. We hope that this paper highlights potential concerns and identifies future avenues for research and development.

In the insects sourced commercially in the UK, high manganese concentrations were observed only in the termites (*Odontotermes* spp. but labelled as *Nasutitermes* spp.). Given the small quantity these insects are sold in (10 g per packet) it is unlikely to be a serious risk if single packets are consumed. Nonetheless, this finding highlights the principal that every species (and form) of insect should be examined thoroughly for nutritional quality and safety before market. There is also considerable variation between the remaining six commercially purchased edible insects in all other minerals examined. Thus, whilst insects can be a source of important micronutrients, targeted analysis of each species will reveal which insects are rich in particular micronutrients. This information is essential for food safety and marketing.

### Conclusions

Alate termites from multiple locations and multiple genera contained high concentrations of Mn. Even small quantities of termites would far exceed the current upper recommended intakes for both adults and children. Results suggest this is biologically derived accumulation, rather than a result of environmental contamination. These are pertinent results considering how widely alates are consumed. We recommend further research to determine the mechanisms for this accumulation and to establish how widespread high Mn concentrations are across termite species and feeding groups. Information about the bioavailability of Mn from edible termites, and how Mn could interact with other dietary minerals during digestion is also lacking and should be a research priority. More generally, our findings highlight the importance of treating insect species on an individual basis when considering using or marketing them as human food.

## Supplementary information

Supplementary tables for manuscript.

## Data Availability

The AAS data will be made available to the dryad repository.

## References

[1] Defoliart GR (1997). An overview of the role of edible insects in preserving biodiversity. Ecology of Food and Nutrition.

[2] Jongema, W. *List of edible insect species of the world. Waganingen, Laboratory of Entomology*. Retrieved from, www.ent.wur. nl/UK/Edible+ insects/Worldwide+species+list/, (2017).

[3] Illgner P, Nel E (2010). The geography of Edible Insects in Sub-Saharan Africa: A study of the mopane caterpillar. The Geographical Journal.

[4] FAO. *Edible insects: Future prospects for food and feed security*. Retrieved from, http://www.fao.org/docrep/018/i3253e/i3253e00.htm (2013).

[5] van Huis, A., & Oonincx, D. G. A. The environmental sustainability of insects as food and feed. A review. *Agronomy for Sustainable Development*, **37****;**10.1007/s13593-017-0452-8 (2017).

[6] Payne CLR, Scarborough P, Rayner M, Nonaka K (2016). A systematic review of nutrient composition data available for 12 commercially available insects, and comparison with reference values. Trends in Food Science and Technology.

[7] Rumpold BA, Schluter OK (2013). Nutritional composition and safety aspects of edible insects. Molecular Nutrition and Food Research.

[8] Banjo A, Aina S (2013). & Salau, A. Shelf Life and Heavy Metals Study of Two Common Edible Insects in Ijebu Division, Southwestern, Nigeria. Journal of Biology and Life Science.

[9] Greenfield R, Akala N, van der Bank F (2012). Heavy Metal Concentrations in Two Populations of Mopane Worms (Imbrasia belina) in the Kruger National Park Pose a Potential Human Health Risk. Bulletin of Environmental Contamination and Toxicology.

[10] European Union. *Regulation (EU) 2015/2283 on novel foods*. Retrieved from, https://eur-lex.europa.eu/legal-content/EN/TXT/?uri=CELEX32015R2283 (2015).

[11] van Huis A (2017). Cultural significance of termites in sub-Saharan Africa. Journal of Ethnobiology and Ethnomedicine.

[12] Figueirêdo, Rozzanna Esther Cavalcanti Reis de, Alexandre Vasconcellos, Iamara Silva Policarpo, and Rômulo Romeu Nóbrega Alves. “Edible and Medicinal Termites: A Global Overview.” Journal of Ethnobiology and Ethnomedicine 11, no. 29 (April 30, 2015). 10.1186/s13002-015-0016-4. 10.1186/s13002-015-0016-4 PMC4427943 25925503

[13] Amadi E, Kiin-kabari D (2016). Nutritional Composition and Microbiology of Some Edible Insects Commonly Eaten in Africa, Hurdles and Future Prospects: A Critical Review. Journal of Food: Microbiology, Safety & Hygiene.

[14] Igwe C, Ujuwondu C, Nwaogu L, Okwu G (2011). Chemical Analysis of an Edible African Termite, Macrotermes nigeriensis; a Potential Antidote to Food Security Problem. Biochemistry and Analytical Biochemistry.

[15] Kinyuru, J. N., G. M. Kenji, and M. S. Njoroge. “Process Development, Nutrition and Sensory Qualities of Wheat Buns Enriched with Edible Termites (Macrotermes Subhylanus) from Lake Victoria Region, Kenya.” African Journal of Food, Agriculture, Nutrition and Development 9, no. 8 (2009). 10.4314/ajfand.v9i8.48411.

[16] Kinyuru JN (2013). Journal of Food Composition and Analysis Nutrient composition of four species of winged termites consumed in western Kenya. Journal of Food Composition and Analysis.

[17] Siulapwa, N, A Mwambungu, E Lungu, and W Sichilima. “Nutritional Value of Four Common Edible Insects in Zambia.” International Journal of Science and Research 3, no. 6 (June 2014): 876–84.

[18] Payne CLR (2015). The mineral composition of five insects as sold for human consumption in Southern Africa. African Journal of Biotechnology.

[19] van der Steen JJM, Cornelissen B, Blacquière T (2016). Think regionally, act locally: metals in honeybee workers in the Netherlands (surveillance study 2008). Environmental Monitoring and Assessment.

[20] Food and Nutrition Board, I. of M. *Dietary Reference Intakes for Calcium, Phosphorus, Magnesium, Vitamin D, and Fluoride*. (S. C. on the S. E. of D. R. Intakes., Ed.). National Academies Press, Washington DC. Retrieved from https://www.ncbi.nlm.nih.gov/books/NBK109825/ (1997). 23115811

[21] Food and Nutrition Board, I. of M. Dietary reference intakes for Vitamin A, Vitamin K, Arsenic, Boron, Chromium, Copper, Iodine, Iron, Manganese, Molybdenum, Nickel, Silicon, Vanadium and Zinc. National Academies Press, Washington DC. Retrieved from, https://www.ncbi.nlm.nih.gov/books/NBK222332/ (2000). 25057538

[22] Riggi LG, Veronesi M, Goergen G, Macfarlane C, Verspoor RL (2016). Observations of entomophagy across Benin – practices and potentials. Food Security.

[23] Siozios, S., Massa, A., Parr C.L., Verspoor, R.L., Hurst, G.D.D. DNA Barcoding reveals incorrect labelling of insects sold as food in the UK. PeerJ, 8, e8496: 10.7717/peerj.8496 10.7717/peerj.8496 PMC7020814 32095344

[24] Fredrikson M, Carlsson NG, Almgren A, Sandberg AS (2002). Simultaneous and sensitive analysis of Cu, Ni, Zn, Co, Mn, and Fe in food and biological samples by ion chromatography. Journal of Agricultural and Food Chemistry.

[25] R Development Core Team, R. R: A Language and Environment for Statistical Computing. (R. D. C. Team, Ed.) *R Foundation for Statistical Computing*. R Foundation for Statistical Computing. 10.1007/978-3-540-74686-7 (2011).

[26] Oibiokpa F, Akanya H, Jigam A, Saidu M (2017). Nutrient and Antinutrient Compositions of Some Edible Insect Species in Northern Nigeria. Fountain Journal of Natural and Applied Sciences.

[27] Chulu, C. Nutrient Composition of the Termite Macrotermes falciger, collected from Lusaka district, a potential agent against malnutrition. masters thesis. The University of Zambia. Retrieved from, http://dspace.unza.zm:8080/xmlui/bitstream/handle/123456789/4388/FinalDissertation.pdf?sequence=1&isAllowed=ymbia-99921.html(2015).

[28] O’Neal SL, Zheng W (2015). Manganese Toxicity Upon Overexposure: A Decade in Review Manganese Toxicity Upon Overexposure: a Decade in Review. Current Environmental Health Report.

[29] Treinen KA, Gray TIMJB, Blazak WF (1995). Developmental Toxicity of Mangafodipir Trisodium and Manganese Chloride in Sprague-Dawley Rats. Teratology.

[30] Fitsanakis VA, Zhang N, Garcia S, Aschner M (2010). Manganese (Mn) and iron (Fe): Interdependency of transport and regulation. Neurotoxicity Research.

[31] Ljung K, Vahter M (2007). Time to re-evaluate the guideline value for manganese in drinking water?. Environmental Health Perspectives.

[32] Wasserman G (2005). Water manganese exposure and children’s intellectual function in Araihazar, Bangladesh. Environmental Health Perspectives.

[33] Finley JW, Monroe P (1997). Mn Absorption: The use of CACO-2 cells as a model of the intestinal epithelia. Nutritional Biochemistry.

[34] Latunde-Dada GO, Yang W, Aviles MV (2016). *In vitro* iron availability from insects and sirloin beef. Journal of agricultural and food chemistry.

[35] Ben-Shahar, Y. The Impact of Environmental Mn Exposure on Insect Biology. Frontiers in Genetics 9. 10.3389/fgene.2018.00070. (2018) 10.3389/fgene.2018.00070 PMC5837978 29545824

[36] Nowak V, Persijn D, Rittenschobber D, Charrondiere R (2016). Review of food composition for edible insects. Food Chemistry.

[37] Cribb BW (2006). Insect mandibles — comparative mechanical properties and links with metal incorporation. Naturwissenschaften.

[38] Mujuru, F. M., Kwiri, R., Nyambi, C., Winini, C., & Moyo, D. N. Microbiological quality of Gonimbrasia belina processed under different traditional practices in Gwanda, Zimbabwe. International Journal of Current Microbiology and Applied Sciences, **3**, 1085–1094; Retrieved from https://www.ijcmas.com/vol-3-9/FelixM.Mujuru,et al.pdf (2014).

[39] Hofrichter M (2002). Review: Lignin conversion by manganese peroxidase (MnP). Enzyme and Microbial Technology.

[40] Johnson R, Thomas R, Wood T, Swift M (1981). The inoculation of the fungus comb in newly founded colonies of some species of the Macrotermitinae (Isoptera) from Nigeria. Journal of Natural History.

[41] Korb J, Aanen D (2003). The evolution of uniparental transmission of fungal symbionts in fungus growing termites (Macrotermitinae). Behavioural Ecology and Sociobiology.

[42] Dobermann D, Swift JA, Field LM (2017). Opportunities and hurdles of edible insects for food and feed. Nutrition Bulletin.

[43] European Food Safety Authority. *General Food Law Regulation* (No. The Regulation (EC) No 178/2002). (2002).

[44] Fanzo J (2015). Ethical issues for human nutrition in the context of global food security and sustainable development. Global Food Security.

[45] Fafchamps, M. *Rural poverty, risk and development*. Edward Elgar Publishing. (2003).

[46] Harrison GW, Humphrey SJ, Verschoor A (2009). Choice under uncertainty: evidence from Ethiopia, India and Uganda. The Economic Journal.

