# Supplementary material for: Mineral analysis reveals extreme manganese concentrations in wild harvested and commercially available edible termites
Source: Sci Rep. 2020 Apr 9;10:6146. doi: 10.1038/s41598-020-63157-7 (PMC7145797; doi:10.1038/s41598-020-63157-7)
Supplement: Supplementary file 1 — Supplementary tables for manuscript. [file 41598_2020_63157_MOESM1_ESM.pdf]

Title: Mineral analysis reveals extreme manganese concentrations in wild harvested and commercially available edible termites.

List of authors

*Rudi L VERSPOOR[1]

Murielle SOGLO[2]

Razack ADEOTI[2]

Rousseau DJOUAKA[2]

Sam EDWARDS[1]

Rikard FRISTEDT[3]

Maud LANGTON[4]

Rosana MORIANA[4]

Matthew OSBORNE[5]

Catherine L. PARR[6,7,8]

Kathryn POWELL[1]

Gregory DD Hurst[1]

Rikard LANDBERG[3]

[1] Institute of integrative Biology, University of Liverpool, Liverpool, United Kingdom L69 7ZB

[2] IITA research station, Godomey, Benin

[3] Chalmers University of Technology, Department of Biology and Biological Engineering, Division of Food and Nutrition Science, SE-412 96 Göteborg, Sweden

[4] Swedish University of Agricultural Sciences, Department of Molecular Sciences, Box 7015, 750 07 Uppsala

[5] Stockholm Environment Institute, Stockholm, Sweden

[6] School of Environmental Science, University of Liverpool, Liverpool, United Kingdom L69 3GP

[7] Department of Zoology & Entomology, University of Pretoria, Pretoria, South Africa

[8] School of Animal, Plant and Environmental Sciences, University of Witwatersrand, Wits, South Africa

***corresponding author,** email = Verspoor@liverpool.ac.uk, telephone = 00447875333238

Supplementary table 1. Showing the co-ordinates of the collection areas for the wild harvested termites from Benin and South Africa. The details of the supplier of commercial insects purchased in the UK are also included. *Species as identified by supplier.

**Location**	**Insect species**	**Type**	**Co-ordinates**
Tanguieta, Benin	*Macrotermes subhyalinus.*	Wild harvested	Area surrounding
	Brachytrupes membranaceus	Wild harvested	10°36'40"N, 1°17'08"E
Parakou, Benin	*Macrotermes* spp.	Wild harvested	
Acornhoek, South Africa	*Odontotermes* spp.	Wild harvested	24°33'58"S, 31°05'54"E
	Macrotermes spp.	Wild harvested	24°34'50.6"S 31°06'56.3"E
Crunchy critters, UK*	*Nasutitermes costalis*	Online purchase	NA
	Locusta migratoria	Online purchase	NA
	Acheta domesticus	Online purchase	NA
	Tenebrio molitor	Online purchase	NA
	Lethoserus indicus	Online purchase	NA
	Atta spp.	Online purchase	NA
	Bombyx mori	Online purchase	NA

Supplementary table 2. Estimates of mineral concentrations for a selection of minerals using 3 independent methods (ICP-MS = Inductively coupled plasma mass spectrometry (carried out by the Swedish National Food Agency), AAS = Atomic Absorption Spectroscopy, IC = Ion Chromatography). The analyses were carried out for a sub-set of termite samples from North West Benin and from Crunchy critters.

Sample	Method	Al	Mn	Fe	Cu	Zn	Mo	Cd	Pb
C.Critters Termites	ICP-MS	4.8 ± 2.6	337.9 ± 50.7	12.3 ± 2.0	4.9 ± 0.7	13.3 ± 1.6	0.08 ± 0.01	0.03 ± 0.01	0.03 ± 0.01
Benin Termites A	ICP-MS	4.6 ± 2.5	564.6 ± 84.7	13.7 ± 2.2	5.2 ± 7.8	9.2 ± 1.1	0.06 ± 0.01	< 0.006	< 0.015
Benin Termites B	ICP-MS	5.1 ± 2.7	323.7 ± 48.6	14.0 ± 2.2	5.5 ± 0.8	9.6 ± 1.2	0.09 ± 0.02	< 0.006	0.05 ± 0.01
Benin Termites C	ICP-MS	11.7 ± 6.2	418.4 ± 62.8	12.9 ± 2.1	4.8 ± 0.7	9.2 ± 1.1	0.08 ± 0.02	< 0.006	0.03 ± 0.01
Benin Termites D	ICP-MS	6.6 ± 3.5	637.7 ± 95.7	15.0 ± 2.4	6.8 ± 1.0	9.7 ± 1.2	0.07 ± 0.01	< 0.006	0.03 ± 0.01
Benin Termites E	ICP-MS	10.4 ± 5.5	503.1 ± 75.5	18.1 ± 2.9	4.9 ± 0.7	10.9 ± 1.3	0.09 ± 0.02	< 0.006	0.1 ± 0.03
C.Critters Termites	AAS	N.A.	271.4 ± 51.6	13.4 ± 0.8	7.6 ± 0.6	12.9 ± 0.3	N.A.	N.A.	N.A.
Benin Termites A	AAS		524.3 ± 127.7	11.4 ± 0.4	7.4 ± 0.6	9.1 ± 0.1			
Benin Termites B	AAS		342.7 ± 32.2	12.7 ± 2.5	8.5 ± 0.4	9.7 ± 0.6			
Benin Termites C	AAS		375.7 ± 82.7	12.8 ± 5.3	6.9 ± 1.0	9.1 ± 0.1			
Benin Termites D	AAS		472.0 ± 43.6	15.2 ± 2.9	9.3 ± 0.2	10.2 ± 0.6			
Benin Termites E	AAS		309.2 ± 164.4	14.6 ± 4.1	6.6 ± 1.1	10.5 ± 0.6			
Benin Termites A	IC	N.A.	481.5 ± 90.4	16.6 ± 0.6	4.2 ± 0.4	6.9 ± 1.2	N.A.	N.A.	N.A.
Benin Termites B	IC		330.6 ± 76.0	15.6 ± 1.1	4.6 ± 0.3	7.1 ± 1.7			
Benin Termites C	IC		359.9 ± 53.0	18.8 ± 2.1	3.4 ± 0.5	5.6 ± 0.8			
Benin Termites D	IC		495.7 ± 87.5	19.6 ± 0.5	4.5 ± 0.8	7.0 ± 1.0			
Benin Termites E	IC		301.2 ± 33.1	20.6 ± 0.5	3.5 ± 0.0	8.6 ± 0.7			

Supplementary Table 4. Composition in wt% of the total elemental content for additional metallic elements examined using scanning electron microscopy for mandibles, abdominal cuticle, spiracle and interior abdomen of *Macrotermes subhyalinus* specimen from Benin.

Element	*mandibles*	*abdominal cuticle*	*spiracle*	*interior abdomen*
***Ni** (%)*	n.d	2	n.d	0.4
***Ti** (%)*	n.d	2.5	n.d	0.7
***Va** (%)*	n.d	3.9	n.d	n.d
***Co** (%)*	n.d	2.7	n.d	n.d
***As** (%)*	n.d	n.d	n.d	0.4
***Al** (%)*	5.0	3	3.9	1.3
***Br** (%)*	n.d	n.d	n.d	n.d
***Cr** (%)*	n.d	n.d	6	n.d
***Si** (%)*	2.6	n.d	2.6	n.d
***P** (%)*	9.7	7.1	9.5	25.8
***S** (%)*	3.0	3.3	2.4	0.5
***Cl** (%)*	24.1	18.8	19	n.d
***K** (%)*	30.8	31.9	33.7	9.1
***Ca** (%)*	2.1	12.2	3.6	49.8
***Na** (%)*	8.5	5	4.6	0.4
